# Retraction: Structural characterization of *Mesobuthus martensii* Karsch peptides and anti-inflammatory potency evaluation in human vascular endothelial cells

**DOI:** 10.1039/d2ra90042d

**Published:** 2022-04-27

**Authors:** Laura Fisher

**Affiliations:** Royal Society of Chemistry Thomas Graham House, Science Park, Milton Road Cambridge CB4 0WF UK advances-rsc@rsc.org

## Abstract

Retraction of ‘Structural characterization of *Mesobuthus martensii* Karsch peptides and anti-inflammatory potency evaluation in human vascular endothelial cells’ by Man Zheng *et al.*, *RSC Adv.*, 2019, **9**, 19365–19374, https://doi.org/10.1039/C9RA01609K.

The Royal Society of Chemistry hereby wholly retracts this *RSC Advances* article due to a significant amount of unattributed text overlap with articles by different author groups, including articles published in *Acta Pharmacologica Sinica* by Yu-ping Zhu *et al.*^1^ and *RSC Advances* by Zhuo Liu *et al.*^2^

Jie Liu opposes the retraction. The other authors have been informed but have not responded to any correspondence regarding the retraction.

Signed: Laura Fisher, Executive Editor, *RSC Advances*

Date: 29^th^ March 2022

## Supplementary Material
